# Clinical Outcomes in Patients with Cystic Fibrosis Receiving CFTR Modulators: A Comparison of Childhood Versus Adolescent Initiation

**DOI:** 10.3390/children12020157

**Published:** 2025-01-28

**Authors:** Eman A. Toraih, Hassan A. Malik, Rahib K. Islam, Humza A. Pirzadah, Ahmed Abdelmaksoud, Rami M. Elshazli, Paul Antwi Boasiako, Shehab Ahmed Alenazi, Angelique Dabel, Jessan A. Jishu, Bandar T. Alenezi, Hani Aiash, Manal S. Fawzy

**Affiliations:** 1 Department of Surgery, School of Medicine, Tulane University, New Orleans, LA 70112, USA; aabelma@medsch.ucr.edu (A.A.); relshazli@tulane.edu (R.M.E.); 2 Department of Cardiovascular Perfusion, Interprofessional Research, College of Health Professions, SUNY Upstate Medical University, New York, NY 13210, USA; 3 Medical Genetics Unit, Department of Histology and Cell Biology, Suez Canal University, Ismailia 41522, Egypt; 4 Louisiana State University Health Sciences Center, New Orleans School of Medicine, New Orleans, LA 70112, USA; hmali1@lsuhsc.edu (H.A.M.); rislam@lsuhsc.edu (R.K.I.); hpirza@lsuhsc.edu (H.A.P.); 5 Department of Biochemistry and Molecular Genetics, Faculty of Physical Therapy, Horus University, New Damietta 34517, Egypt; 6 Department of Biological Sciences, Faculty of Science, New Mansoura University, New Mansoura City 35742, Egypt; 7 College of Health Professions, SUNY Upstate Medical University, New York, NY 13210, USA; antwibop@upstate.edu (P.A.B.); dabela@upstate.edu (A.D.); 8 Department of Pediatrics, Faculty of Medicine, Northern Border University, Arar 91431, Saudi Arabia; shehab.alenazi@nbu.edu.sa; 9 School of Medicine, Tulane University, New Orleans, LA 70112, USA; jjishu@tulane.edu; 10 Department of Pharmacology, Faculty of Medicine, Northern Border University, Arar 91431, Saudi Arabia; bandar.alenezi@nbu.edu.sa; 11 SUNY Upstate Medical University, New York, NY 13210, USA; aiashh@upstate.edu; 12 Center for Health Research, Northern Border University, Arar 91431, Saudi Arabia; manal.darwish@nbu.edu.sa

**Keywords:** genetic inheritance, targeted therapy, global research network, multicenter, healthcare burden

## Abstract

Background/objectives: Cystic fibrosis (CF) is a life-limiting genetic disorder affecting multiple organ systems. This study compared clinical outcomes, hospitalization rates, and survival between children and adolescents with CF who received CFTR modulator therapies (ivacaftor, lumacaftor, tezacaftor, and elexacaftor). Methods: A retrospective cohort study was conducted using data from the TriNetX global collaborative network. Patients with CF aged 2–12 years (children) and 13–18 years (adolescents) who received CFTR modulator therapies were included. The propensity score matching balanced baseline characteristics between the two age groups. Results: After propensity score matching, 946 patients per group were analyzed. The incidence of respiratory failure (3.81% vs. 1.06%, *p* < 0.001) and respiratory infections (62.7% vs. 57.5%, *p* = 0.021) were significantly higher in adolescents compared to children. Adolescents had a higher risk of respiratory failure (HR = 3.6, 95% CI = 1.79–7.21, *p* < 0.001) and respiratory infections (HR = 1.09, 95% CI = 1.01–1.17, *p* < 0.001). Adolescents also had a higher hospitalization rate (29.6% vs. 20.3%, *p* < 0.001), with a 47% higher risk (HR = 1.47, 95% CI = 1.22–1.77, *p* = 0.001), more hospital visits per person (8.8 vs. 3.7, *p* = 0.004), and longer hospital stays (32.7 vs. 20.4 days, *p* = 0.006). Mortality rates were similar between the groups (1.58% vs. 1.26%, *p* = 0.56). Conclusions: CF patients who initiated CFTR modulator therapies during adolescence had a higher incidence of respiratory failure, respiratory infections, hospitalization rates, and healthcare resource utilization compared to those who started therapy in childhood, despite similar mortality rates. These findings highlight the importance of the early initiation of CFTR modulator therapies.

## 1. Introduction

Cystic fibrosis (CF) is a life-threatening autosomal recessive disorder with a prevalence of 1 in 2700 births worldwide [1]. It is caused by mutations in the cystic fibrosis transmembrane conductance regulator (CFTR) gene. The dysfunction of the CFTR protein leads to the accumulation of thick, sticky mucus in various organ systems, primarily the lungs and digestive tract [2,3]. Patients with CF often experience progressive lung damage, recurrent respiratory infections, pancreatic insufficiency, and other complications. These manifestations significantly impact patients’ quality of life and life expectancy, making CF a major global health concern [3,4]. CF can occur through various mutations, each of which causes impairment of the CFTR protein’s ability to function adequately [3,5,6]. The CFTR protein is an ion channel that transports chloride across the cell membrane. Dysfunction in the CFTR protein results in it either being trapped in the endoplasmic reticulum of the cell and not reaching the cell membrane or reaching the cell membrane but not being able to function properly or sufficiently [5,7,8,9,10]. Both defects result in impaired ability to regulate chloride transport across the cell.

In the lungs, sinuses, pancreas, GI tract, biliary, and hepatic systems, the CFTR protein normally transports chloride from the intracellular to extracellular space [3,5]. Sodium and water subsequently follow the chloride and are excreted out of the cell, allowing these organ systems to maintain an adequately low viscosity of secretions in which flow is not impeded [5,7,8,9,10]. In CF, the cell cannot sufficiently excrete chloride, and therefore neither sodium nor water, resulting in thick secretions in the pulmonary system, GI tract, exocrine pancreas, biliary system, hepatic system, and sinuses [3,7]. The thick, highly viscous secretions cause stagnation and permit bacterial growth, causing a significantly increased risk of infection in any of these systems, especially the pulmonary system [8,9]. The impaired secretions of the GI tract, exocrine pancreas, and biliary system also cause fat malabsorption, leading to fat-soluble vitamin deficiencies in patients [7]. In sweat glands, the direction of the CFTR protein is reversed, so it functions to absorb chloride, and subsequently sodium and water, from the skin [3,5]. Impairment of the CFTR protein here results in the inability of CF patients to sufficiently reabsorb sodium and water, predisposing them to dehydration and salt depletion. This defect of CFTR in the sweat glands is also responsible for the pathognomonic salty skin in CF patients [2,11,12,13].

Overall, the impairment of the CFTR protein in CF results in thick secretions, ultimately causing a significantly increased risk for a variety of infections, fat malabsorption, and electrolyte depletion [3,7]. CFTR dysfunction in sweat glands results in elevated salt levels in the sweat and insufficient chloride and bicarbonate secretion, leading to thick mucus and a cycle of mucus obstruction, chronic infection, and inflammation. This pathophysiological process underlies the multisystemic nature of CF, affecting not only the lungs but also the digestive system, reproductive tract, and other organs [3,7].

**Figure 1:**
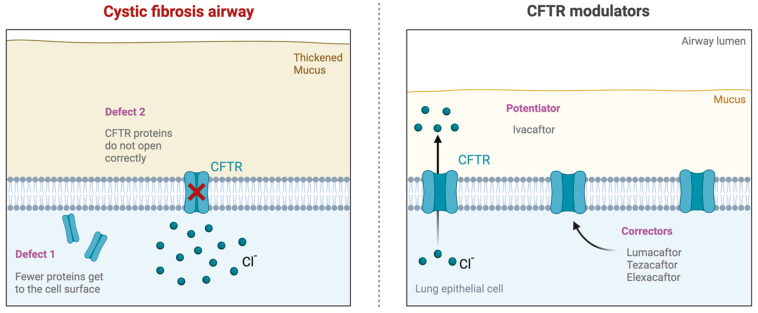
Impact of CFTR modulator therapy. (**Left**) Cystic fibrosis transmembrane conductance regulator (CFTR) mutations cause CF dysfunction, leading to thick mucus, chronic infection, and inflammation. (**Right**) Novel targeted CFTR modulator therapies. Ivacaftor potentiates the channel open probability of the CFTR protein at the cell surface. Lumacaftor, tezacaftor, and elexacaftor work to improve cellular processing and trafficking by allowing for more CFTR proteins to come to the cell surface.

Traditionally, CF treatment focused on alleviating symptoms and managing complications using mucolytics, antibiotics, and pancreatic enzyme replacement therapy [3,7,14]. However, recent advancements in CF treatment have led to the development of CFTR modulator therapies, which aim to correct the underlying protein defect [3,15]. These innovative therapies, including ivacaftor, lumacaftor, tezacaftor, and elexacaftor, have shown promising results in improving lung function, reducing pulmonary exacerbations, and enhancing the overall well-being of CF patients (Figure 1) [3,10,16,17]. CFTR modulators have differing mechanisms of action based on the sequence of protein formation or function they target. Ivacaftor functions as a “potentiator” that increases the time CFTR channels in the cell membrane remain open [12,13,16,17,18,19]. Lumacaftor and tezecaftor are “correctors” that function by correcting CFTR misprocessing due to an amino acid deletion, allowing for improved CFTR localization to the cell membrane and, therefore, increased density at the plasma membrane [12,16,17,20,21]. Elexacaftor is a next-generation corrector that follows the same mechanism as lumacaftor and tezecaftor [12,16,17,22]. These three correctors are typically combined with an ivacaftor for maximum efficacy. Altogether, CFTR modulators represent a new class of drugs that partially restore the activity of the CFTR by improving the production, intracellular processing, trafficking, and/or function of the defective CFTR protein.

Despite the remarkable progress in CF treatment, the comparative effectiveness and safety of CFTR modulators across different age groups remain unclear. Moreover, the long-term impact of these treatments on critical outcomes, such as lung transplantation and mortality, has not been extensively studied, with recent literature indicating the need to study the effects of long-term modulator use and early initiation of therapy [11]. Understanding the differential effects of CFTR modulators in various pediatric population subgroups is crucial for optimizing treatment strategies and improving clinical decision-making.

Given the potential differences in disease progression and treatment response between children and adolescents with CF, it is essential to investigate the clinical outcomes and effectiveness of CFTR modulator therapies across different age groups. The timing of intervention initiation may play a critical role in determining long-term outcomes, as early treatment may prevent or slow the irreversible damage caused by CF. However, the optimal age for initiating CFTR modulator therapy and its impact on disease progression and survival remain to be elucidated. To address these knowledge gaps, this study aims to compare the clinical outcomes, hospitalization rates, and survival of children (aged 2–12 years) and adolescents (aged 13–18 years) with CF who received CFTR modulator therapies using real-world data from a large, multi-national patient cohort.

## 2. Materials and Methods

### 2.1. Study Design and Data Source

This retrospective cohort study utilized data from the TriNetX global collaborative network (https://trinetx.com/) (accessed on 25 May 2024), which includes de-identified electronic health records (EHRs) from 120 healthcare organizations (HCOs) across 19 countries, which includes a total of 145,313,976 patients. The TriNetX platform provides a secure, cloud-based health research platform that enables the analysis of real-world data while maintaining patient privacy and confidentiality.

### 2.2. Study Population

Patients with cystic fibrosis were identified using the International Classification of Diseases, Tenth Revision, Clinical Modification (ICD-10-CM) diagnosis code E84.x. The study included patients diagnosed with cystic fibrosis between 1 January 2010 and 31 December 2022, who were treatment-naive to CFTR modulator therapies at the time of initial prescription (Supplementary Table S1). Patients were excluded if they had a prior history of solid organ transplantation or respiratory failure (Supplementary Table S2).

The study population was stratified into two age groups at the time of the first CFTR modulator prescription: children (aged 2–12 years) and adolescents (aged 13–18 years). Patients were considered to be treated with CFTR modulator drugs if they received their first prescription for one of the following medications (ivacaftor, lumacaftor, tezacaftor, or elexacaftor) during the study period.

### 2.3. Propensity Score Matching

Propensity score matching was performed to minimize potential confounding factors and ensure comparability between the adolescent and child groups. A propensity score was calculated for each patient using a logistic regression model that included 24 baseline characteristics, such as demographics, comorbidities, clinical presentation, and medication use. Patients from the adolescent and child groups were then matched 1:1 using the nearest neighbor matching algorithm with a caliper width of 0.2 standard deviations of the logit of the propensity score.

### 2.4. Outcomes

The primary outcomes of interest were respiratory infections, lung transplantation, respiratory failure, hospitalization rate, hospital visits per person, and hospital length of stay. All-cause mortality was assessed as a secondary outcome (Supplementary Table S3). Outcomes were compared between the matched adolescent and child groups using appropriate statistical tests.

### 2.5. Statistical Analysis

Categorical variables were presented as frequencies and percentages, while continuous variables were presented as means ± standard deviations or medians with interquartile ranges, depending on their distribution. Chi-square tests were used to compare categorical variables, while Student’s *t*-tests or Mann–Whitney U tests were used for continuous variables, as appropriate. Kaplan–Meier survival curves were constructed to compare overall survival between the adolescent and child groups, and the log-rank test was used to assess the difference in survival probabilities. Cox proportional hazard regression was used to calculate hazard ratios (HRs) and 95% confidence intervals (CIs) for all-cause mortality. All statistical analyses were performed using the TriNetX platform (https://trinetx.com/) (accessed on 25 May 2024), and a two-sided *p*-value < 0.05 was considered statistically significant.

## 3. Results

### 3.1. Study Population

**Figure 2:**
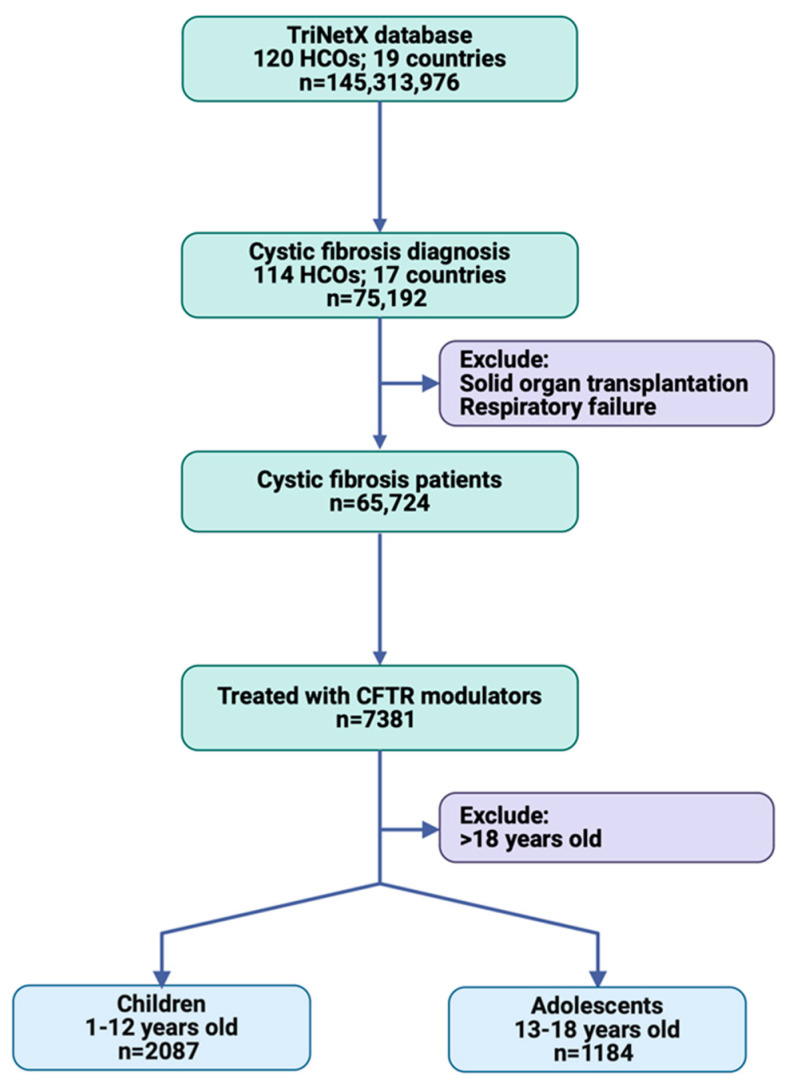
Workflow of cohort selection in TriNetX.

A total of 75,192 patients with cystic fibrosis were identified from 114 HCOs in 17 countries, including patients of any age and sex. After excluding those with solid organ transplantation and respiratory failure, the study population consisted of 65,724 patients. Among these, 7381 patients were treated with one of the four CFTR modulator drugs: ivacaftor, lumacaftor, tezacaftor, or elexacaftor. The study population was further stratified by age, with 2087 patients aged 2–12 years from 48 HCOs and 1184 patients aged 13–18 years from 56 HCOs (Figure 2).

### 3.2. Baseline Characteristics

Table 1 presents the characteristics of the pediatric populations. Significant differences (*p* < 0.05) were observed between adolescents and children in several demographic, clinical, and laboratory parameters, as well as medication use.

### 3.3. Propensity Score Matching

**Figure 3:**
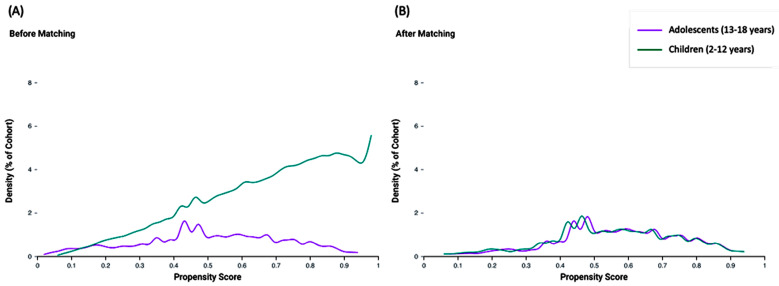
Propensity score density function before (**A**) and after (**B**) matching. Adolescent and child age groups were compared.

Propensity score matching was conducted on a comprehensive set of 24 variables, encompassing key aspects of patient demographics, clinical characteristics, and medication use. The propensity score density functions for the adolescent and child cohorts are depicted before and after matching. Prior to matching, the density functions exhibited notable differences, indicating potential imbalances in baseline characteristics. After propensity score matching, the density functions of the two cohorts aligned closely, demonstrating the effectiveness of the matching process in creating balanced groups for comparison (Figure 3).

After matching, 946 patients per group were included, with a mean age of 15.6 ± 1.8 years for adolescents and 7.5 ± 3.5 years for children. The baseline characteristics of the matched cohorts yielded no significant differences (Table 2), confirming the success of the propensity score matching process in creating comparable groups for subsequent analyses.

### 3.4. Pulmonary Outcomes

In both unmatched and matched cohorts who received CFTR modulator drugs, neither the adolescent nor the child group experienced lung transplantation at some time during their lives. However, the incidence of respiratory failure (3.81% vs. 1.06%, *p* < 0.001) and respiratory infections (62.7% vs. 57.5%, *p* = 0.021) were significantly higher in the adolescent group compared to the child group (Table 3). Delayed intervention was associated with four times the risk of respiratory failure than early intervention before 13 years old (HR: 3.6, 95% CI: 1.79–7.21, *p* < 0.001). In addition, the hazard ratio for respiratory infections was 1.09 (95% CI: 1.01–1.17, *p* < 0.001), suggesting a 9% increased risk for adolescents compared to children.

### 3.5. Hospitalization Rate

The adolescent group experienced a significantly higher hospitalization rate compared to the child group (29.6% vs. 20.3%, *p* < 0.001) (Table 3). The hazard ratio for hospitalization was 1.47 (95% CI: 1.22–1.77, *p* = 0.001), indicating a 47% higher risk of hospitalization for adolescents. Additionally, adolescents had a higher average number of hospital visits per person (8.8 vs. 3.7 times, *p* = 0.004) and a longer average hospital length of stay (32.7 days vs. 20.4 days, *p* = 0.006).

### 3.6. Survival Analysis

**Figure 4:**
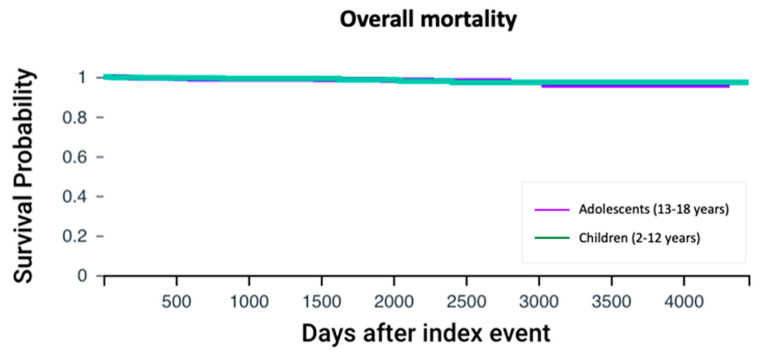
Overall survival analysis. Kaplan–Meier survival curves comparing the overall survival between adolescents and children with cystic fibrosis. The log-rank test was used to assess the difference in survival probabilities between the two groups.

Mortality rates were similar between adolescents and children with cystic fibrosis (1.58% vs. 1.26%, *p* = 0.56). Cox regression analysis did not show differential survival when CFTR modulator therapy was initiated 13 years or older compared to earlier intervention (HR 1.09, 95% CI: 0.51–2.34, *p* = 0.86) (Figure 4).

## 4. Discussion

This large, multi-national retrospective cohort study compared clinical outcomes, hospitalization rates, and survival between children and adolescents with cystic fibrosis (CF) who received CFTR modulator therapies. The results demonstrated that adolescents had a significantly higher incidence of respiratory infections, hospitalization rates, and healthcare resource utilization compared to children despite similar mortality rates.

The higher incidence of respiratory infections in adolescents could be attributed to the progression of lung disease with age and the cumulative effects of chronic inflammation and structural damage [8,16,17,18]. This finding underscores the importance of early intervention with CFTR modulator therapies to prevent or slow the deterioration of lung function and reduce the risk of respiratory infections. In doing so, early initiation through these therapies may help preserve lung health and improve long-term outcomes in patients with CF [9,16,17,18,23,24,25]. Additionally, CF has an impact on psychological and physical development, especially in adolescents. Chronic illness can separate patients from their peers and create an environment of isolation. CF also impacts biological development in several aspects, including short stature due to malabsorption, lower self-esteem, and social impairment due to increased fatigue [19]. CFTR modulators can mitigate these effects for patients and early use can potentially reduce the physical and mental burden of adolescent CF patients [26].

The significantly higher hospitalization rate, more hospital visits per person, and longer hospital stays observed in adolescents suggest that the disease burden and healthcare resource utilization increase as CF patients transition from childhood to adolescence [9,16,17,18,27]. It has been reported that the use of CFTR modulators in children has decreased hospitalization, emergency department visits, and respiratory exacerbation and has increased lung function and quality of life among children [17,18,28]. This increase in adolescent hospitalization could be due to the complex interplay of factors such as hormonal changes, treatment adherence challenges, and psychosocial issues that emerge during adolescence [19]. Addressing these challenges through comprehensive, age-appropriate care and support can improve overall outcomes and reduce adverse effects for adolescents with CF.

Although mortality rates were similar between the groups, the trend towards an increased risk of mortality when CFTR modulator therapy was initiated in adolescence is concerning. While this finding did not reach statistical significance, likely due to the relatively short follow-up period and the low overall mortality rate in this young population, it highlights the potential long-term impact of delaying CFTR modulator therapy initiation. This observation reinforces the importance of early intervention [9,16,17,18,23] and the need for long-term studies to fully understand the impact of treatment timing on mortality in CF patients.

The strengths of this study include the large, diverse patient population from multiple countries and the use of propensity score matching to balance baseline characteristics between the age groups. This approach helps to minimize potential confounding factors and provides a more accurate comparison of outcomes between children and adolescents with CF. However, the study also has some limitations that should be considered when interpreting the results. First, the retrospective nature of the study may introduce potential biases and confounding factors that cannot be fully accounted for. Second, the lack of detailed clinical data, including pulmonary function tests and imaging diagnostics, such as chest X-rays or CT scans, as well as the molecular profiles such as CFTR mutation types, and detailed genotype information, limits the ability to fully characterize disease severity and treatment response across the spectrum of CF genotypes. This limits our ability to fully assess the influence of specific CFTR gene mutations on treatment response and may affect the precision and generalizability of our analysis. Incorporating these data in future studies would provide a more comprehensive understanding of the impact of CFTR modulator therapies on specific patient subgroups. Third, while our study included patients on any CFTR modulator to maintain statistical power and reflect real-world treatment patterns, future studies with larger sample sizes could examine outcomes stratified by specific modulator types, particularly as longer-term data becomes available in stable patient cohorts. Finally, the relatively short follow-up period may not capture the long-term impact of CFTR modulator therapies on clinical outcomes and survival. Longer-term studies are needed to assess the full potential of these therapies in improving the lives of patients with CF.

## 5. Conclusions

In conclusion, this study provides valuable insights into the real-world clinical outcomes and healthcare resource utilization of children and adolescents with CF who received CFTR modulator therapies. The findings highlight the importance of early initiation of CFTR modulator therapies and the need for close monitoring and management of CF patients transitioning from childhood to adolescence. Addressing the unique challenges faced by adolescents with CF through comprehensive, age-appropriate care and support may help optimize treatment outcomes and improve overall quality of life. Future studies with longer follow-up periods and more detailed clinical data are needed to further elucidate the long-term impact of CFTR modulator therapies on pediatric CF populations and to inform clinical decision-making and patient management strategies.

## Figures and Tables

**Table 1:** Characteristics of study groups.

Characteristics	Levels	Adolescents (*n* = 1184)	Children (*n* = 2087)	*p*-Value
Demographics				
Age at onset	Mean ± SD, years	15.7 ± 1.8	7.0 ± 3.4	**<0.001**
Sex	Female	607 (51.3%)	989 (47.4%)	**0.033**
	Male	576 (48.6%)	1096 (52.5%)	
Race	White	0.98	1791 (85.8%)	
	Black	26 (2.2%)	82 (3.9%)	
	Other race	27 (2.3%)	58 (2.8%)	
Ethnicity	Not Hispanic or Latino	924 (78%)	1745 (83.6%)	0.76
	Hispanic or Latino	79 (6.7%)	145 (6.9%)	
Clinical data				
Comorbidities	Diabetes mellitus	219 (18.5%)	83 (4%)	**<0.001**
	Diseases of liver	135 (11.4%)	136 (6.5%)	**<0.001**
	Gastro-esophageal reflux disease	299 (25.3%)	741 (35.5%)	**<0.001**
	Vitamin D deficiency	287 (24.2%)	463 (22.2%)	0.18
	Other vitamin deficiencies	75 (6.3%)	107 (5.1%)	0.15
Presentation	Exocrine pancreatic insufficiency	284 (24%)	772 (37%)	**<0.001**
	Cystic fibrosis with pulmonary manifestations	812 (68.6%)	1447 (69.3%)	0.65
	Cystic fibrosis with intestinal manifestations	509 (43%)	1117 (53.5%)	**<0.001**
	Cystic fibrosis with other manifestations	493 (41.6%)	908 (43.5%)	0.30
	Sodium chloride in sweat ≥ 60 mmol/L	681 (57.5%)	1368 (65.5%)	**<0.001**
Lab data				
Complete blood picture	Blood leukocytes, 10^3^/uL	8.7 ± 3.6	9.1 ± 3.4	**0.014**
	Lymphocytes/100 leukocytes, %	29.5 ± 10.7	42.3 ± 14.8	**<0.001**
	Neutrophils, 10^3^/uL	142.1 ± 1019.1	89.4 ± 627.6	0.17
	Eosinophils/100 leukocytes, %	2.9 ± 2.8	2.6 ± 2.6	0.07
Glycemia	Hemoglobin A1c, %	5.8 ± 1.5	5.5 ± 1.0	**<0.001**
Liver function test	Total bilirubin, mg/dL	0.5 ± 0.3	0.3 ± 0.3	**<0.001**
	Serum albumin, g/dL	4.2 ± 0.5	4.3 ± 0.4	**<0.001**
	Aspartate aminotransferase, U/L	28.5 ± 18.6	35.5 ± 15.6	**<0.001**
	Alanine aminotransferase, U/L	31.7 ± 24.9	31.3 ± 20.9	0.69
	Alkaline phosphatase, U/L	183.3 ± 105.3	256.7 ± 85.5	**<0.001**
Renal function test	Blood urea nitrogen, mg/dL	12.8 ± 5.8	12.4 ± 3.9	0.09
	Serum creatinine, mg/dL	0.7 ± 0.4	0.5 ± 3.0	0.24
Electrolytes	Sodium, mmol/L	139.4 ± 2.4	139.0 ± 2.1	**0.002**
	Potassium, mmol/L	4.2 ± 0.4	4.2 ± 0.5	0.05
	Chloride, mmol/L	103.9 ± 3.0	104.2 ± 5.5	0.25
	Bicarbonate, mmol/L	25.0 ± 2.8	23.4 ± 2.6	**<0.001**
Inflammatory markers	C-reactive protein, mh/L	15.6 ± 22.9	15.3 ± 30.1	0.92
	Erythrocyte sedimentation rate, mm/h	17.8 ± 19.2	13.0 ± 15.4	**0.005**
Medications				
Respiratory medications	Antiasthma/bronchodilators	847 (71.5%)	1752 (83.9%)	**<0.001**
	Nasal anti-inflammatories	630 (53.2%)	1106 (53%)	0.91
	Adrenal corticosteroids	537 (45.4%)	1059 (50.7%)	**0.003**
	Nasal decongestants	115 (9.7%)	212 (10.2%)	0.68
	Nasal antihistamines	48 (4.1%)	64 (3.1%)	0.14
	Antitussives/expectorants	59 (5%)	42 (2%)	**<0.001**
	Mucolytics	34 (2.9%)	70 (3.4%)	0.45
Digestive drugs	Digestants	696 (58.8%)	1506 (72.2%)	**<0.001**
Nutritional supplements	Therapeutic nutrients/minerals/electrolytes	715 (60.4%)	1431 (68.6%)	**<0.001**
	Vitamins	571 (48.2%)	1043 (50%)	0.34

Data are presented as number (percentage) or mean ± standard deviation (SD). Two-sided Chi-square or Student’s *t* tests were used. Bold values are significant at *p* < 0.05.

**Table 2:** Characteristics of matched cohorts.

Characteristics	Levels	Adolescents (*n* = 946)	Children (*n* = 946)	*p*-Value
Demographics				
Sex	Female	462 (48.8%)	487 (51.5%)	0.25
	Male	483 (51.1%)	458 (48.4%)	
Race	White	809 (85.5%)	815 (86.2%)	0.69
	Black	26 (2.7%)	22 (2.3%)	
	Other race	22 (2.3%)	28 (3%)	
Ethnicity	Not Hispanic or Latino	732 (77.4%)	755 (79.8%)	0.19
	Hispanic or Latino	60 (6.3%)	81 (8.6%)	
Clinical data				
Comorbidities	Diabetes mellitus	72 (7.6%)	76 (8%)	0.73
	Diseases of liver	85 (9%)	84 (8.9%)	0.94
	Gastro-esophageal reflux disease	228 (24.1%)	238 (25.2%)	0.59
	Vitamin D deficiency	208 (22%)	200 (21.1%)	0.66
	Other vitamin deficiencies	54 (5.7%)	42 (4.4%)	0.21
Presentation	Exocrine pancreatic insufficiency	242 (25.6%)	212 (22.4%)	0.11
	Cystic fibrosis with pulmonary manifestations	620 (65.5%)	631 (66.7%)	0.59
	Cystic fibrosis with intestinal manifestations	404 (42.7%)	425 (44.9%)	0.33
	Cystic fibrosis with other manifestations	352 (37.2%)	374 (39.5%)	0.30
	Sodium chloride in sweat ≥ 60 mmol/L	539 (57%)	563 (59.5%)	0.26
Medications				
Respiratory medications	Antiasthma/bronchodilators	687 (72.6%)	696 (73.6%)	0.64
	Nasal anti-inflammatories	491 (51.9%)	511 (54%)	0.36
	Adrenal corticosteroids	422 (44.6%)	439 (46.4%)	0.43
	Nasal decongestants	83 (8.8%)	90 (9.5%)	0.58
	Nasal antihistamines	41 (4.3%)	33 (3.5%)	0.34
	Antitussives/expectorants	35 (3.7%)	31 (3.3%)	0.62
	Mucolytics	25 (2.6%)	26 (2.7%)	0.89
Digestive drugs	Digestants	546 (57.7%)	599 (63.3%)	0.13
Nutritional supplements	Therapeutic nutrients/minerals/electrolytes	566 (59.8%)	586 (61.9%)	0.35
	Vitamins	437 (46.2%)	420 (44.4%)	0.43

Data are presented as numbers (percentages). A two-sided Chi-square test was used.

**Table 3:** Outcomes comparison between adolescents and children with cystic fibrosis.

Outcomes	Adolescents (*n* = 946)	Children (*n* = 946)	*p*-Value
Lung transplantation	0	0	NA
Respiratory failure	36 (3.81%)	10 (1.06%)	**<0.001**
Respiratory infections	593 (62.7%)	544 (57.5%)	**0.021**
Hospitalization rate	280 (29.6%)	192 (20.3%)	**<0.001**
Hospital visits per person	8.8 ± 23.9 (median = 3)	3.77 ± 8.1 (median = 2)	**0.004**
Hospital length of stay, days	32.7 ± 55.5 (median = 16)	20.4 ± 36.9 (median = 10)	**0.006**
All-cause mortality	15 (1.58%)	12 (1.26%)	0.56

Data are presented as numbers (percentages) or mean ± standard deviation (SD). Two-sided Chi-square or Student’s *t*-tests were used for categorical variables, and Mann–Whitney U tests were used for continuous variables. Bold values are significant at *p* < 0.05.

## Data Availability

Restrictions apply to the availability of these data due to. Data were obtained from the TriNetX database and are available at (https://trinetx.com/) (accessed on 25 May 2024) with the permission of the TriNetX database authority.

## Supplementary Materials

The following supporting information can be downloaded at: https://www.mdpi.com/article/10.3390/children12020157/s1, Table S1: Inclusion codes; Table S2: Exclusion codes; Table S3: Outcome codes.

## References

[1] Zampoli M., Morrow B.M., Paul G. (2023). Real-world disparities and ethical considerations with access to CFTR modulator drugs: Mind the gap!. Front. Pharmacol..

[2] King J.A., Nichols A.-L., Bentley S., Carr S.B., Davies J.C. (2022). An Update on CFTR Modulators as New Therapies for Cystic Fibrosis. Pediatr. Drugs.

[3] Mariotti Zani E., Grandinetti R., Cunico D., Torelli L., Fainardi V., Pisi G., Esposito S. (2023). Nutritional Care in Children with Cystic Fibrosis. Nutrients.

[4] Alenazi S.A. (2019). Cystic fibrosis: Saudi arabia current situation and perspectives. Ann. Clin. Anal. Med..

[5] Hodson M., Bush A., Geddes D. (2012). Cystic Fibrosis.

[6] Khan M.A., Ali Z.S., Sweezey N., Grasemann H., Palaniyar N. (2019). Progression of Cystic Fibrosis Lung Disease from Childhood to Adulthood: Neutrophils, Neutrophil Extracellular Trap (NET) Formation, and NET Degradation. Genes.

[7] Sellers Z.M. (2020). Pancreatic complications in children with cystic fibrosis. Curr. Opin. Pediatr..

[8] Turcios N.L. (2020). Cystic Fibrosis Lung Disease: An Overview. Respir. Care.

[9] Ong T., Ramsey B.W. (2023). Cystic Fibrosis: A Review. JAMA.

[10] Burgener E.B., Moss R.B. (2018). Cystic fibrosis transmembrane conductance regulator modulators: Precision medicine in cystic fibrosis. Curr. Opin. Pediatr..

[11] Dwight M., Marshall B. (2021). CFTR modulators: Transformative therapies for cystic fibrosis. J. Manag. Care Spec. Pharm..

[12] Kuek S., McCullagh A., Paul E., Armstrong D. (2023). Real world outcomes of CFTR modulator therapy in Australian adults and children. Pulm. Pharmacol. Ther..

[13] Ramsey B.W., Davies J., McElvaney N.G., Tullis E., Bell S.C., Dřevínek P., Griese M., McKone E.F., Wainwright C.E., Konstan M.W. (2011). A CFTR Potentiator in Patients with Cystic Fibrosis and the G551D Mutation. N. Engl. J. Med..

[14] Kuhn R.J., Nahata M.C. (1985). Therapeutic management of cystic fibrosis. Clin. Pharm..

[15] Choong E., Sauty A., Koutsokera A., Blanchon S., André P., Decosterd L. (2022). Therapeutic Drug Monitoring of Ivacaftor, Lumacaftor, Tezacaftor, and Elexacaftor in Cystic Fibrosis: Where Are We Now?. Pharmaceutics.

[16] Wainwright C., McColley S.A., McNally P., Powers M., Ratjen F., Rayment J.H., Retsch-Bogart G., Roesch E., Ahluwalia N., Chin A. (2023). Long-Term Safety and Efficacy of Elexacaftor/Tezacaftor/Ivacaftor in Children Aged ≥ 6 Years with Cystic Fibrosis and at Least One F508del Allele: A Phase 3, Open-Label Clinical Trial. Am. J. Respir. Crit. Care Med..

[17] Mall M.A., Brugha R., Gartner S., Legg J., Moeller A., Mondejar-Lopez P., Prais D., Pressler T., Ratjen F., Reix P. (2022). Efficacy and Safety of Elexacaftor/Tezacaftor/Ivacaftor in Children 6 Through 11 Years of Age with Cystic Fibrosis Heterozygous for F508del and a Minimal Function Mutation: A Phase 3b, Randomized, Placebo-controlled Study. Am. J. Respir. Crit. Care Med..

[18] Aoyama B.C., Mogayzel P.J. (2020). Ivacaftor for the treatment of cystic fibrosis in children under six years of age. Expert Rev. Respir. Med..

[19] Segal T.Y. (2008). Adolescence: What the cystic fibrosis team needs to know. J. R. Soc. Med..

[20] Taylor-Cousar Jennifer L., Munck A., McKone Edward F., van der Ent Cornelis K., Moeller A., Simard C., Wang Linda T., Ingenito Edward P., McKee C., Lu Y. (2017). Tezacaftor–Ivacaftor in Patients with Cystic Fibrosis Homozygous for Phe508del. N. Engl. J. Med..

[21] Wainwright C.E., Elborn J.S., Ramsey B.W., Marigowda G., Huang X., Cipolli M., Colombo C., Davies J.C., De Boeck K., Flume P.A. (2015). Lumacaftor–Ivacaftor in Patients with Cystic Fibrosis Homozygous for Phe508del CFTR. N. Engl. J. Med..

[22] Middleton Peter G., Mall Marcus A., Dřevínek P., Lands Larry C., McKone Edward F., Polineni D., Ramsey Bonnie W., Taylor-Cousar Jennifer L., Tullis E., Vermeulen F. (2019). Elexacaftor–Tezacaftor–Ivacaftor for Cystic Fibrosis with a Single Phe508del Allele. N. Engl. J. Med..

[23] Allen L., Allen L., Carr S.B., Davies G., Downey D., Egan M., Forton J.T., Gray R., Haworth C., Horsley A. (2023). Future therapies for cystic fibrosis. Nat. Commun..

[24] Muilwijk D., Bierlaagh M., van Mourik P., Kraaijkamp J., van der Meer R., van den Bor R., Heijerman H., Eijkemans R., Beekman J., van der Ent K. (2021). Prediction of Real-World Long-Term Outcomes of People with CF Homozygous for the F508del Mutation Treated with CFTR Modulators. J. Pers. Med..

[25] Muilwijk D., Zomer-van Ommen D.D., Gulmans V.A.M., Eijkemans M.J.C., van der Ent C.K. (2022). Long-term effectiveness of dual CFTR modulator treatment of cystic fibrosis. ERJ Open Res..

[26] Kramer-Golinkoff E., Camacho A., Kramer L., Taylor-Cousar J.L. (2022). A survey: Understanding the health and perspectives of people with CF not benefiting from CFTR modulators. Pediatr. Pulmonol..

[27] van Gool K., Norman R., Delatycki M.B., Hall J., Massie J. (2013). Understanding the costs of care for cystic fibrosis: An analysis by age and health state. Value Health.

[28] Marshall L.Z., Espinosa R., Starner C.I., Gleason P.P. (2023). Real-world outcomes and direct care cost before and after elexacaftor/tezacaftor/ivacaftor initiation in commercially insured members with cystic fibrosis. J. Manag. Care Spec. Pharm..

