# Supplementary material for: Clinical Outcomes in Patients with Cystic Fibrosis Receiving CFTR Modulators: A Comparison of Childhood Versus Adolescent Initiation
Source: Children (Basel). 2025 Jan 28;12(2):157. doi: 10.3390/children12020157 (PMC11854606; doi:10.3390/children12020157)
Supplement: Supplementary file 1 [file children-12-00157-s001.zip › children-3408026-supplementary.pdf]

Table S1. Inclusion codes.

Category	Code	Content
Confirmed Cystic fibrosis diagnosis		
Diagnosis	UMLS:ICD10CM:E84	Cystic fibrosis
Diagnosis	UMLS:ICD10CM:E84.9	Cystic fibrosis, unspecified
Diagnosis	UMLS:ICD10CM:Z14.1	Cystic fibrosis carrier
Diagnosis	UMLS:ICD10CM:E84.0	Cystic fibrosis with pulmonary manifestations
Diagnosis	UMLS:ICD10CM:E84.8	Cystic fibrosis with other manifestations
Diagnosis	UMLS:ICD10CM:E84.11	Meconium ileus in cystic fibrosis
Diagnosis	UMLS:ICD10CM:E84.1	Cystic fibrosis with intestinal manifestations
Diagnosis	UMLS:ICD10CM:E84.19	Cystic fibrosis with other intestinal manifestations
Laboratory	UMLS:LNC:2077-6	Chloride [Moles/volume] in Sweat (at least 60.00 mmol/L (most recent))
CFTR modulators		
Medication	NLM:RXNORM:1243041	Ivacaftor (2-12 or 13-18 years old at event)
Medication	NLM:RXNORM:1655922	Lumacaftor (2-12 or 13-18 years old at event)
Medication	NLM:RXNORM:1999382	Tezacaftor (2-12 or 13-18 years old at event)
Medication	NLM:RXNORM:2256951	Elexacaftor (2-12 or 13-18 years old at event)

Table S2. Exclusion codes.

Category	Code	Content
Exclude prior solid organ transplantation		
Procedure	UMLS:SNOMED:77465005	Transplantation
Procedure	UMLS:ICD10PCS:0TY	Transplantation
Procedure	UMLS:ICD10PCS:0FY	Transplantation
Procedure	UMLS:ICD10PCS:02Y	Transplantation
Procedure	UMLS:ICD10PCS:0BY	Transplantation
Procedure	UMLS:ICD10PCS:10Y	Transplantation
Procedure	UMLS:ICD10PCS:0UY	Transplantation
Procedure	UMLS:ICD10PCS:0DY	Transplantation
Procedure	UMLS:ICD10PCS:07Y	Transplantation
Procedure	UMLS:ICD10PCS:0WY	Transplantation
Procedure	UMLS:ICD10PCS:0XY	Transplantation
Procedure	UMLS:ICD10PCS:0TY00Z0	Transplantation of Right Kidney, Allogeneic, Open Approach
Procedure	UMLS:CPT:1021134	Transplantation and Post-Transplantation Cellular Infusion Procedures on the Hemic and Lymphatic Systems
Procedure	UMLS:ICD10PCS:0TY10Z0	Transplantation of Left Kidney, Allogeneic, Open Approach
Procedure	UMLS:ICD10PCS:0FY00Z0	Transplantation of Liver, Allogeneic, Open Approach
Procedure	UMLS:ICD10PCS:0TY00Z1	Transplantation of Right Kidney, Syngeneic, Open Approach
Procedure	UMLS:ICD10PCS:0TY10Z1	Transplantation of Left Kidney, Syngeneic, Open Approach
Procedure	UMLS:ICD10PCS:0TY00Z2	Transplantation of Right Kidney, Zooplastic, Open Approach
Procedure	UMLS:ICD10PCS:0TY10Z2	Transplantation of Left Kidney, Zooplastic, Open Approach
Procedure	UMLS:SNOMED:82316003	Transplantation of abdominal tissue
Procedure	UMLS:ICD10PCS:02YA0Z0	Transplantation of Heart, Allogeneic, Open Approach
Procedure	UMLS:ICD10PCS:0FY00Z1	Transplantation of Liver, Syngeneic, Open Approach
Procedure	UMLS:ICD10PCS:0FY00Z2	Transplantation of Liver, Zooplastic, Open Approach
Procedure	UMLS:ICD10PCS:0BYM0Z0	Transplantation of Bilateral Lungs, Allogeneic, Open Approach
Procedure	UMLS:ICD10PCS:0FYG0Z0	Transplantation of Pancreas, Allogeneic, Open Approach
Procedure	UMLS:ICD10PCS:02YA0Z1	Transplantation of Heart, Syngeneic, Open Approach
Procedure	UMLS:ICD10PCS:02YA0Z2	Transplantation of Heart, Zooplastic, Open Approach
Procedure	UMLS:SNOMED:18027006	Transplantation of liver

Procedure	UMLS:CPT:48554	Transplantation of pancreatic allograft
Procedure	UMLS:ICD10PCS:0BYM0Z1	Transplantation of Bilateral Lungs, Syngeneic, Open Approach
Procedure	UMLS:ICD10PCS:0BYM0Z2	Transplantation of Bilateral Lungs, Zooplastic, Open Approach
Procedure	UMLS:ICD10PCS:0BYL0Z0	Transplantation of Left Lung, Allogeneic, Open Approach
Procedure	UMLS:ICD10PCS:0BYK0Z0	Transplantation of Right Lung, Allogeneic, Open Approach
Procedure	UMLS:ICD10PCS:0FYG0Z1	Transplantation of Pancreas, Syngeneic, Open Approach
Procedure	UMLS:ICD10PCS:0BYK0Z1	Transplantation of Right Lung, Syngeneic, Open Approach
Procedure	UMLS:ICD10PCS:0BYL0Z2	Transplantation of Left Lung, Zooplastic, Open Approach
Procedure	UMLS:ICD10PCS:0BYK0Z2	Transplantation of Right Lung, Zooplastic, Open Approach
Procedure	UMLS:ICD10PCS:0BYL0Z1	Transplantation of Left Lung, Syngeneic, Open Approach
Procedure	UMLS:ICD10PCS:0BYD0Z1	Transplantation of Right Middle Lung Lobe, Syngeneic, Open Approach
Procedure	UMLS:ICD10PCS:0BYJ0Z0	Transplantation of Left Lower Lung Lobe, Allogeneic, Open Approach
Procedure	UMLS:ICD10PCS:0BYJ0Z1	Transplantation of Left Lower Lung Lobe, Syngeneic, Open Approach
Procedure	UMLS:ICD10PCS:0BYJ0Z2	Transplantation of Left Lower Lung Lobe, Zooplastic, Open Approach
Procedure	UMLS:ICD10PCS:0BYG0Z0	Transplantation of Left Upper Lung Lobe, Allogeneic, Open Approach
Procedure	UMLS:ICD10PCS:0BYG0Z1	Transplantation of Left Upper Lung Lobe, Syngeneic, Open Approach
Procedure	UMLS:ICD10PCS:0BYG0Z2	Transplantation of Left Upper Lung Lobe, Zooplastic, Open Approach
Procedure	UMLS:ICD10PCS:0BYH0Z0	Transplantation of Lung Lingula, Allogeneic, Open Approach
Procedure	UMLS:ICD10PCS:0BYH0Z1	Transplantation of Lung Lingula, Syngeneic, Open Approach
Procedure	UMLS:ICD10PCS:0BYH0Z2	Transplantation of Lung Lingula, Zooplastic, Open Approach
Procedure	UMLS:ICD10PCS:0BYF0Z0	Transplantation of Right Lower Lung Lobe, Allogeneic, Open Approach
Procedure	UMLS:ICD10PCS:0BYF0Z1	Transplantation of Right Lower Lung Lobe, Syngeneic, Open Approach
Procedure	UMLS:ICD10PCS:0BYF0Z2	Transplantation of Right Lower Lung Lobe, Zooplastic, Open Approach
Procedure	UMLS:ICD10PCS:0BYD0Z0	Transplantation of Right Middle Lung Lobe, Allogeneic, Open Approach
Procedure	UMLS:ICD10PCS:0BYD0Z2	Transplantation of Right Middle Lung Lobe, Zooplastic, Open Approach

Procedure	UMLS:ICD10PCS:0BYC0Z0	Transplantation of Right Upper Lung Lobe, Allogeneic, Open Approach
Procedure	UMLS:ICD10PCS:0BYC0Z1	Transplantation of Right Upper Lung Lobe, Syngeneic, Open Approach
Procedure	UMLS:ICD10PCS:0BYC0Z2	Transplantation of Right Upper Lung Lobe, Zooplastic, Open Approach
Procedure	UMLS:SNOMED:32413006	Transplantation of heart
Procedure	UMLS:ICD10PCS:0FYG0Z2	Transplantation of Pancreas, Zooplastic, Open Approach
Procedure	UMLS:SNOMED:6471000179103	Transplantation of kidney and pancreas
Procedure	UMLS:CPT:32854	Lung transplant, double (bilateral sequential or en bloc); with cardiopulmonary bypass
Procedure	UMLS:CPT:32853	Lung transplant, double (bilateral sequential or en bloc); without cardiopulmonary bypass
Exclude prior respiratory failure before CFTR modulator intake		
Diagnosis	UMLS:ICD10CM:J96	Respiratory failure, not elsewhere classified
Diagnosis	UMLS:ICD10CM:J96.00	Acute respiratory failure, unspecified whether with hypoxia or hypercapnia
Diagnosis	UMLS:ICD10CM:J96.9	Respiratory failure, unspecified
Diagnosis	UMLS:ICD10CM:J96.91	Respiratory failure, unspecified with hypoxia
Diagnosis	UMLS:ICD10CM:J96.92	Respiratory failure, unspecified with hypercapnia
Diagnosis	UMLS:ICD10CM:J96.10	Chronic respiratory failure, unspecified whether with hypoxia or hypercapnia
Diagnosis	UMLS:ICD10CM:J96.0	Acute respiratory failure
Diagnosis	UMLS:ICD10CM:J96.1	Chronic respiratory failure
Diagnosis	UMLS:ICD10CM:J96.20	Acute and chronic respiratory failure, unspecified whether with hypoxia or hypercapnia
Diagnosis	UMLS:ICD10CM:J96.01	Acute respiratory failure with hypoxia
Diagnosis	UMLS:ICD10CM:J96.02	Acute respiratory failure with hypercapnia
Diagnosis	UMLS:ICD10CM:J96.11	Chronic respiratory failure with hypoxia
Diagnosis	UMLS:ICD10CM:J96.12	Chronic respiratory failure with hypercapnia
Diagnosis	UMLS:ICD10CM:J96.2	Acute and chronic respiratory failure
Diagnosis	UMLS:ICD10CM:J96.21	Acute and chronic respiratory failure with hypoxia
Diagnosis	UMLS:ICD10CM:J96.22	Acute and chronic respiratory failure with hypercapnia
Diagnosis	UMLS:ICD10CM:J96.90	Respiratory failure, unspecified, unspecified whether with hypoxia or hypercapnia
Diagnosis	UMLS:ICD10CM:R09.2	Respiratory arrest
Diagnosis	UMLS:ICD10CM:Z99.1	Dependence on respirator

Table S3. Outcome codes.

Category	Code	Content
All-cause mortality		
Demographics	Deceased	Deceased
Diagnosis	UMLS:ICD10CM:R99	Ill-defined and unknown cause of mortality
Lung transplantation		
Procedure	UMLS:ICD10PCS:0BYM0Z1	Transplantation of Bilateral Lungs, Syngeneic, Open Approach
Procedure	UMLS:ICD10PCS:0BYM0Z2	Transplantation of Bilateral Lungs, Zooplastic, Open Approach
Procedure	UMLS:ICD10PCS:0BYL0Z0	Transplantation of Left Lung, Allogeneic, Open Approach
Procedure	UMLS:ICD10PCS:0BYK0Z0	Transplantation of Right Lung, Allogeneic, Open Approach
Procedure	UMLS:ICD10PCS:0BYK0Z1	Transplantation of Right Lung, Syngeneic, Open Approach
Procedure	UMLS:ICD10PCS:0BYL0Z2	Transplantation of Left Lung, Zooplastic, Open Approach
Procedure	UMLS:ICD10PCS:0BYK0Z2	Transplantation of Right Lung, Zooplastic, Open Approach
Procedure	UMLS:ICD10PCS:0BYL0Z1	Transplantation of Left Lung, Syngeneic, Open Approach
Procedure	UMLS:ICD10PCS:0BYD0Z1	Transplantation of Right Middle Lung Lobe, Syngeneic, Open Approach
Procedure	UMLS:ICD10PCS:0BYJ0Z0	Transplantation of Left Lower Lung Lobe, Allogeneic, Open Approach
Procedure	UMLS:ICD10PCS:0BYJ0Z1	Transplantation of Left Lower Lung Lobe, Syngeneic, Open Approach
Procedure	UMLS:ICD10PCS:0BYJ0Z2	Transplantation of Left Lower Lung Lobe, Zooplastic, Open Approach
Procedure	UMLS:ICD10PCS:0BYG0Z0	Transplantation of Left Upper Lung Lobe, Allogeneic, Open Approach
Procedure	UMLS:ICD10PCS:0BYG0Z1	Transplantation of Left Upper Lung Lobe, Syngeneic, Open Approach
Procedure	UMLS:ICD10PCS:0BYG0Z2	Transplantation of Left Upper Lung Lobe, Zooplastic, Open Approach
Procedure	UMLS:ICD10PCS:0BYH0Z0	Transplantation of Lung Lingula, Allogeneic, Open Approach
Procedure	UMLS:ICD10PCS:0BYH0Z1	Transplantation of Lung Lingula, Syngeneic, Open Approach
Procedure	UMLS:ICD10PCS:0BYH0Z2	Transplantation of Lung Lingula, Zooplastic, Open Approach
Procedure	UMLS:ICD10PCS:0BYF0Z0	Transplantation of Right Lower Lung Lobe, Allogeneic, Open Approach
Procedure	UMLS:ICD10PCS:0BYF0Z1	Transplantation of Right Lower Lung Lobe, Syngeneic, Open Approach
Procedure	UMLS:ICD10PCS:0BYF0Z2	Transplantation of Right Lower Lung Lobe, Zooplastic, Open Approach
Procedure	UMLS:ICD10PCS:0BYD0Z0	Transplantation of Right Middle Lung Lobe, Allogeneic, Open Approach

Procedure	UMLS:ICD10PCS:0BYD0Z2	Transplantation of Right Middle Lung Lobe, Zooplastic, Open Approach
Procedure	UMLS:ICD10PCS:0BYC0Z0	Transplantation of Right Upper Lung Lobe, Allogeneic, Open Approach
Procedure	UMLS:ICD10PCS:0BYC0Z1	Transplantation of Right Upper Lung Lobe, Syngeneic, Open Approach
Procedure	UMLS:ICD10PCS:0BYC0Z2	Transplantation of Right Upper Lung Lobe, Zooplastic, Open Approach
Respiratory failure		
Diagnosis	UMLS:ICD10CM:J96	Respiratory failure, not elsewhere classified
Diagnosis	UMLS:ICD10CM:J96.00	Acute respiratory failure, unspecified whether with hypoxia or hypercapnia
Diagnosis	UMLS:ICD10CM:J96.9	Respiratory failure, unspecified
Diagnosis	UMLS:ICD10CM:J96.91	Respiratory failure, unspecified with hypoxia
Diagnosis	UMLS:ICD10CM:J96.92	Respiratory failure, unspecified with hypercapnia
Diagnosis	UMLS:ICD10CM:J96.0	Acute respiratory failure
Diagnosis	UMLS:ICD10CM:J96.20	Acute and chronic respiratory failure, unspecified whether with hypoxia or hypercapnia
Diagnosis	UMLS:ICD10CM:J96.01	Acute respiratory failure with hypoxia
Diagnosis	UMLS:ICD10CM:J96.02	Acute respiratory failure with hypercapnia
Diagnosis	UMLS:ICD10CM:J96.2	Acute and chronic respiratory failure
Diagnosis	UMLS:ICD10CM:J96.21	Acute and chronic respiratory failure with hypoxia
Diagnosis	UMLS:ICD10CM:J96.22	Acute and chronic respiratory failure with hypercapnia
Diagnosis	UMLS:ICD10CM:J96.90	Respiratory failure, unspecified, unspecified whether with hypoxia or hypercapnia
Respiratory infection		
Diagnosis	UMLS:ICD10CM:J00-J06	Acute upper respiratory infections
Diagnosis	UMLS:ICD10CM:J09-J18	Influenza and pneumonia
Diagnosis	UMLS:ICD10CM:J40-J4A	Chronic lower respiratory diseases
Hospitalization visits and days		
Procedure	UMLS:CPT:1013659	Hospital Inpatient and Observation Care Services
Procedure	UMLS:SNOMED:32485007	Hospital admission
Procedure	UMLS:SNOMED:50699000	Hospital admission, short-term
